# Intra- and Inter-Reader Variations in Lung Nodule Measurements: Influences of Nodule Size, Location, and Observers

**DOI:** 10.3390/diagnostics12102319

**Published:** 2022-09-26

**Authors:** Hong Chen, Haozhe Huang, Jianye Zhang, Xuexue Wang, Mengyang Han, Chanjun Ding, Jinhong Wang

**Affiliations:** 1 Department of Medical Imaging, Shanghai Mental Health Center, Shanghai Jiao Tong University School of Medicine, Shanghai 200030, China; 2 Department of Interventional Radiology, Fudan University Shanghai Cancer Center, 270 Dongan Road, Xuhui District, Shanghai 200032, China; 3 Department of Oncology, Shanghai Medical College, Fudan University, 130 Dongan Road, Xuhui District, Shanghai 200032, China

**Keywords:** chest, computed tomography, pulmonary nodules, measurement, imaging, three-dimensional

## Abstract

(1) Background: Accurate measurement of lung-nodule size is necessary, but whether a three-dimensional volume measurement is better or more reliable than the one-dimensional method is still unclear. This study aimed to investigate the intra- and inter-reader variations according to nodule type, size, three-dimensional volume measurements, and one-dimensional linear measurements. (2) Methods: This retrospective study included computed tomography (CT) examinations of lung nodules and volume measurements performed from October to December 2016. Two radiologists independently performed all measurements. Intra-class correlation coefficients (ICC) and Bland-Altman plots were used for analysis. (3) Results: The overall variability in the calculated volume was larger than when using the semiautomatic volume measurement. Nodules <6 mm tended to have larger variability than nodules ≥6 mm in both one-dimensional and calculated volume measurements. The isolated type showed smaller variability in both intra- and inter-reader comparisons. The juxta-vascular type showed the largest variability in both one-dimensional and calculated volume measurements. The variability was decreased when using the 3D volume semiautomated software. (4) Conclusions: The present study suggests that 3D semiautomatic volume measurements showed lower variability than the calculated volume measurement. Nodule size and location influence measurement variability. The intra- and inter-reader variabilities in nodule volume measurement were considerable.

## 1. Introduction

Lung cancer is the most harmful malignant tumor in public health, with a high incidence and serious disease burden [1]. According to the latest report of the International Cancer Research Institute of the World Health Organization in 2022, the mortality of lung cancer ranks first among malignant tumors and at 2.5 times higher than colorectal cancers, the second leading cause of cancer death [1,2]. The prognosis of lung cancer is closely related to the clinical stage. The 5 year survival rate of patients with early lung cancer can reach 59% after surgery, while it is only 6% for patients with advanced lung cancer after treatment [2]. Therefore, early detection and early diagnosis of lung cancer is the key to the survival and prognosis of patients [3]. At present, low-dose CT, as the primary means of lung cancer screening, has been widely recommended by authoritative clinical guidelines [4,5,6,7,8]. The grading management of pulmonary nodules after screening mainly depends on the size of pulmonary nodules and the changes in size during the follow-up period [8,9,10,11]. Therefore, accurately measuring the diameter and volume of pulmonary nodules is crucial to the development of management and follow-up strategies for pulmonary nodules.

Scanning and reconstruction parameters, nodule shape, lung volume, lung parenchyma on CT images, nodule size, location, readers’ observation, measurement methods, and software can affect nodule size [12,13,14,15], leading to erroneous estimations of the growth and the inaccurate prediction of the nodules. In 1979, the World Health Organization (WHO) first introduced a criterion based on the change in the size of the lesion by determining the largest diameter and its perpendicular length (bidimensional measurement) for each measurable lesion [16]. Later, a study showed that one-dimensional measurements of tumor lesions might better identify the area of tumor killed by an anti-neoplastic agent rather than the bidimensional measurement [17]. These criteria use a one-dimensional measurement instead of a bi-dimensional measurement to determine treatment response [18]. The one-dimensional measurement might fail to detect irregular growth since tumor growth is a three-dimensional (3D) phenomenon, and the growth pattern is not necessarily spherical [19]. The Fleischner Association’s 2017 guidelines recommended that the diameter of pulmonary nodules be expressed as the average of their maximum length diameter and vertical diameter [8]. In 2019, the lung imaging reporting and data system (Lung CT Screening Reporting and Data System, Lung-RADS) 1.1 guidance by the American College of Radiology added the volume of pulmonary nodules to the grading standard and stipulated that the measurement results of all pulmonary nodules’ diameters and volumes should be in mm [20].

Furthermore, with the development of computer-aided diagnosis techniques, 3D measurements are now routinely used in clinical applications. Nevertheless, it remains undefined whether the 3D volumetric measurement of pulmonary nodules is superior to and more reliable than the one-dimensional method when assessing lung nodules. Therefore, this study investigated the intra- and inter-reader variations according to nodule type, size, 3D volumetric measurements, and one-dimensional linear measurements to find an accurate method to measure pulmonary nodules size. The results could help provide a basis for evaluating and managing pulmonary nodules. Then, the structure of our research is presented from the following aspects: Section 2 explains the methodology, Section 3 clarifies the results, Section 4 makes a discussion, and the last section draws conclusions.

## 2. Material and Methods

### 2.1. Study Design and Nodule Selection

This retrospective study included CT examinations of lung nodules. The subjects who underwent CT between October and December 2016 in Shanghai Mental Health Center XX were selected. All eligible lung nodule image sets during this period were examined. Calcified or cavitary nodules and image sets with poor image quality were excluded. The study was approved by the ethics committee.

### 2.2. CT Scanning

All chest CT examinations were performed using a spiral multi-detector CT scanner (GE Healthcare, Waukesha, WI, USA) without contrast. The low-dose screening protocol was used: 120 kVp, 30 effective mAs, and single-breath holding. The scanning range was from the chest entrance to the bottom of the lung, including the entirety of the lungs. The images were reconstructed with a standard lung kernel at a section thickness of 2 mm, without an intersection gap, field of view 350 mm × 350 mm, and matrix 512 × 512, according to the routine screening protocol.

### 2.3. Linear and Volumetric Measurements of the Nodule

Two experienced chest CT radiologists (readers A and B, both with >10 years of experience) were instructed to first identify and measure the lung nodules on the pre-selected images independently. Measurements were performed on the screening CT examinations using the largest diameter of the nodule in the linear unidimensional display. Multiple measurements were repeatedly performed to determine the largest diameter. The largest diameters were used to calculate the volume of the lung nodules using the formula: V = 4/3πr^3^. Then, 3D semiautomatic volume measurements were performed using the 3D display. Reader A repeated both linear unidimensional and volumetric measurements at a 1-week interval while being blinded to the results from the previous measurements. Reader B only made the measurements once. A database was constructed from the linear and volumetric measurements of each nodule.

For 3D semi-automatic volume measurements, the Lung CARE volumetry software (GE Healthcare, Waukesha, WI, USA) was used. The center of the nodule was selected in the axial plane. When the nodule was marked, the initial 3D template originating from the center point was produced in color. The 3D volume of the nodule was automatically obtained. If a nodule touched vessels or the pleura, the first measurement result was not satisfying because the volume of a nodule may not cover the whole nodule or extend to the adjacent vessels or pleura. The readers manually adjusted the included 3D volume by volume decrease or increase according to the coverage of the nodule in the one-dimensional axial plane.

### 2.4. Definitions of Nodules

**Figure 1:**
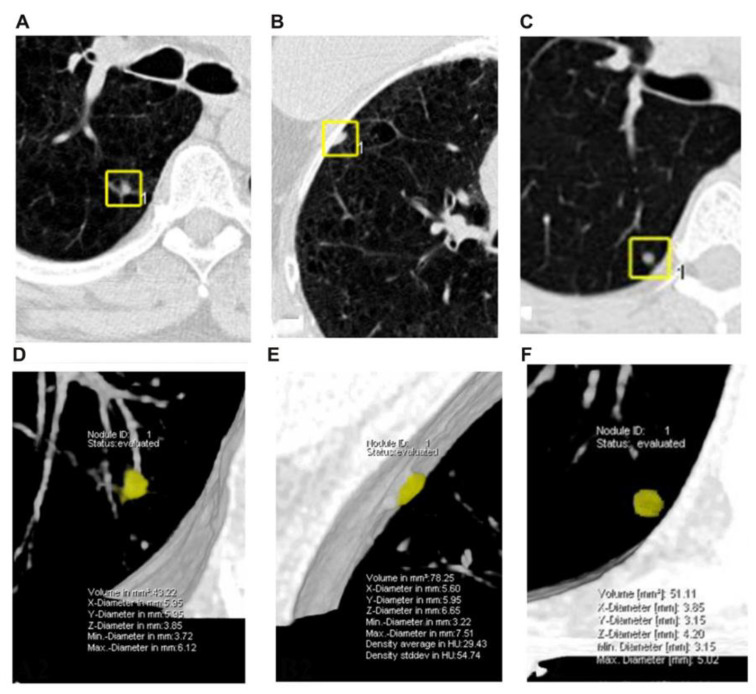
Lung window and 3D display of three types of nodules using the Lung CARE software. (**A**,**D**) are the juxta-vascular type. (**B**,**E**) are the juxta-pleural type. (**C**,**F**) are the isolated nodule type.

Nodules were categorized into three types based on their location within the lungs: isolated, juxta-vascular, and juxta-pleural. Isolated nodules were completely surrounded by aerated lung parenchyma. Juxta-pleural nodules were located within 2 mm of the pleura. Juxta-vascular nodules were sitting on or adjacent to blood vessels (Figure 1). Nodule size was defined as the largest diameter in the CT axial plane. If a nodule was found on several consecutive images, the image depicting the largest diameter was selected. According to the 2017 Fleischner Association guidelines, lung nodules were divided into three groups by size: (1) <6 mm; (2) ≥6 and <8 mm; and (3) ≥8 mm [8]. The guidelines set the evaluation threshold of the nodules as 6 and 8 mm and nodules with a diameter of ≥6 mm should be actively dealt with.

### 2.5. Statistical Analysis

The continuous data were presented as means ± standard deviation or as median (range) according to their distribution, determined using the Kolmogorov-Smirnov test. Categorical data were presented as numbers (percentages). The intra-reader difference was determined using the original and repeated measurements by reader A. The inter-reader difference was derived from the first measurement of reader A, and the measurement of reader B. Measurements were analyzed based on different nodule types and sizes (Figure 1).

The intraclass correlation coefficient (ICC) was used to evaluate the reliability of the measurements. Its value was between 0 and 1. A value of 0 meant untrusted and 1 meant completely trusted. It was generally believed that a reliability coefficient lower than 0.4 indicated poor reliability, while a reliability coefficient greater than 0.75 indicated good reliability. Higher ICC values were needed for quantitative data. A scatter plot was used to explore the distribution law of the two measurements methods of the lung nodules. By observing the distribution of data points on the scatter plot, we could infer the correlation between variables. The Bland-Altman plot was used To further assess the intra- and inter-reader differences [21]. MedCalc software (MedCalc Software bvba, Ostend, Flanders, Belgium) was used for statistical analysis. Two-sided *p*-values <0.05 were considered statistically significant.

## 3. Results

### 3.1. Characteristics of the Patients and Nodules

The CT examinations of 56 patients performed from October to December 2016 were included. There were 29 (51.8%) men and 27 (48.2%) women, and the mean age was 61.7 (21–85) years. There were 83 lung nodules among the 56 patients. The longest diameter range of the nodules was 3.3–18.2 mm. There were 41 (49.4%) nodules <6 mm, 29 (34.9%) nodules of ≥6 and <8 mm, and 13 (15.7%) nodules ≥8 mm. The nodules were categorized as isolated (n = 31), juxta-vascular (n = 19), and juxta-pleural nodules (n = 33). Table 1 shows the descriptive statistics of the measurements obtained by readers A and B.

### 3.2. Intraclass Correlation Coefficients

To explore the reliability between the calculated volume using the longest diameter and 3D semiautomatic volume, Table 2 presents the ICC for the calculated volume using the longest diameter and the 3D semiautomatic volume between different nodule types, different sizes, and different readers. The measurement of all nodules showed good reliability, but there were differences among different nodule types and different sizes. For different nodule sizes, the nodules ≥8 mm showed relatively larger ICC (>0.9) than the nodules with other sizes (<8 mm). For different nodule types, the isolated nodule type showed a relatively greater ICC (>0.9) than the other types.

**Figure 2:**
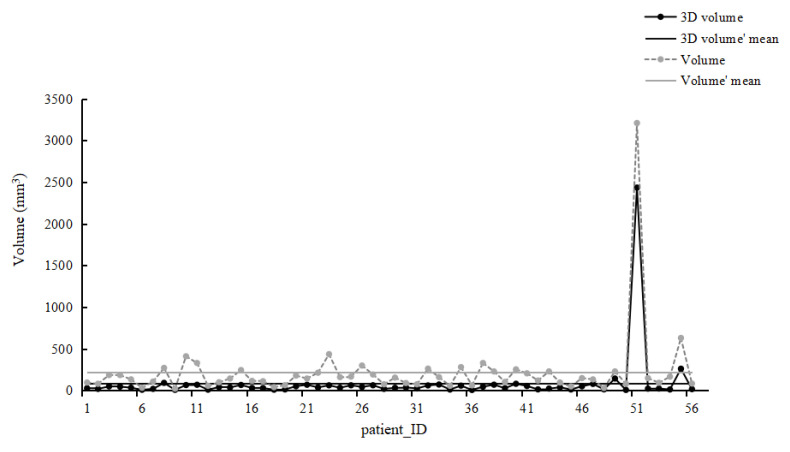
Scatter plots of the two volume measurements. The solid line represents 3D volume, which shows the scatter plot of 56 patients’ 3D semiautomatic volume measurement. The dotted line represents Volume, which shows the scatter plot of 56 patients’ calculated volume using the longest diameter.

Figure 2 shows the distribution law of the two measurement methods of lung nodules. By observing the distribution of data points on the scatter plot, we inferred the correlation between the overall data. The dotted line shows the scatter plot of 56 patients’ calculated volume using the longest diameter. The solid line shows the scatter plot of 56 patients’ 3D semiautomatic volume measurement. The dispersion of values in the solid line is smaller than that in the dotted line, indicating a small degree of variability in the volume of 3D semi-automatic measurement of 56 patients. However, the overall trend of the two measurement methods is similar. The volume obtained by the long diameter measurement is larger than that obtained by the 3D semi-automatic measurement, which may be due to the use of the longest diameter to calculate the volume.

Table 3 presents the ICCs of the intra- and inter-reader measurements based on overall nodules, different sizes, and different types to explore the reliability between different readers and repeated readings. The overall data showed good reliability, but there were differences between different measurement methods. Furthermore, the intra-reader measurements showed greater ICCs than inter-reader measurements, especially in 3D semiautomatic volume measurements between A1 and 2. For different nodule sizes, the nodules ≥8 mm showed relatively larger ICCs than the smaller nodules (<8 mm) in both intra- and inter-reader measurement comparisons. The ICC for the inter-reader calculated volume measurements tended to increase along with the increasing nodule size. For different nodule types, there were no obvious differences in the intra-reader comparison, but the inter-reader 3D measurement of the isolated nodule type showed a relatively greater ICC (1.000) than the other types.

### 3.3. Bland-Altman Plots for All Measurements

Table 4 presents the Bland-Altman plots and correlation results deriving from the overall nodule measurements. In the intra- and inter-reader comparisons, the limits of agreement of the 3D semiautomatic volume measurements were smaller than for the calculated volume measurements. Hence, the inter-reader comparison showed larger limits of agreement than that of the intra-reader comparison.

### 3.4. Bland-Altman Plots Deriving from Different Nodule Size

Table 5 presents the results of the mean differences and limits of agreement for the different nodule types. The limits of agreement of the diameter measurement in nodules <6 mm were larger than in the larger nodules (≥6 mm) in intra- and inter-reader comparisons. Thus, the calculated volume, which was calculated using the longest diameter, followed a similar tendency as the diameter measurements, but it had larger limits of agreement. In the intra-reader comparison, the smallest limit of agreement of 3D semiautomatic volume measurement was observed in nodules <6 mm, but in the inter-reader comparison, the smallest limit of agreement was observed in nodules ≥8 mm.

### 3.5. Bland-Altman Plots Deriving from Different Nodule Types

Table 6 presents the results of the mean differences and limits of agreement deriving from different nodule types. The limits of agreement for the isolated nodule type were smaller than for the other nodule types in intra- and inter-reader comparisons. On the other hand, the juxta-vascular nodule type showed the largest limits of agreement except in the intra-reader 3D volume measurements.

## 4. Discussion

Accurate measurement of lung nodule size is necessary to determine its benign or malignant nature or determine its response to treatment, especially in nodule follow-up management [9,10,11,22,23]. But it remains undefined whether 3D volumetric measurement is superior to or more reliable than the one-dimensional method. Therefore, this study aimed to investigate the contribution of nodule type, size, and different readers to the differences between 3D volumetric and one-dimensional linear measurements of lung nodules. The results suggest that the semiautomatic volume measurements showed lower variability than the calculated volume measurements. However, the measurement of the Juxta-vascular nodules type should be more careful. The type nodules are vulnerable to the influence by vascular shadows, which may lead to measurement deviation (Table 6). Nodule size and location influence measurement variability. The intra-reader and inter-reader variabilities in nodule volume measurements were considerable. In the follow-up management of nodules, it is more likely to be followed up by the same observer to increase the reliability of measurement. Previous studies indicated that the one-dimensional measurement type could replace the more complex bi-dimensional measurement [17,24]. Nevertheless, each measurement has some difficulties in estimating the size of irregular or confluent lesions and discrepancies arise in scan planes and patient positioning [10,25]. Advanced CT technology and new 3D semiautomatic volumetric measurement software are thought to be better methods to quantitatively assess the total tumor volume and associated masses [9,11,14]. The present study was designed thoroughly compare one-dimensional (linear) and 3D semiautomatic volumetric measurements for small lung nodules with large sample size.

The results showed that the overall variation in 3D volumetric measurement was smaller than that of the calculated volume using linear measurement (Figure 2 and Table 4). Each reading and the overall data showed good reliability, which means that both the calculated volume measurement using the longest diameter and the 3D semiautomatic volume measurement is reliable. But the 3D semiautomatic volume measurement showed a smaller degree of variability. The main influencing factors are the non-isolated nodules and smaller-size nodules (<8 mm) (Table 3 and Table 5). In this study, 63% (52 of 83) of the lung nodules were juxta-structure (juxta-vascular or juxta-pleural) nodules, which might be considered irregular to some extent. Linear measurements can hardly estimate the volume of irregular lesions because the nodules vary according to the measurement plane. The nodules are not perfectly spherical, and the adjacent structure deforms or truncates the lesion. Furthermore, a small variation in diameter measurement might cause large volumetric variations when using the formula V = 4/3πr^3^ since the measurement error is exponential and multiplied by 4/3π. On the other hand, the volume obtained using semiautomatic 3D measurements is calculated by counting the number of voxels directly in a nodule. Therefore, those are probably the reasons for the important difference in volume measurement variability between the two methods of nodule volume measurements. A previous study showed that 3D measurements allow an objective evaluation, reducing inter-observer variability in evaluating chemotherapy response of lung tumors [26]. Therefore, the 3D measurement could be superior to the traditional linear method.

The analysis of the nodule size groups showed that smaller nodules had the largest variations in calculated volume measurement. In the intra-reader comparison of 3D volume measurement, the variation of each nodule size was smaller than in the calculated volume measurement, but the variation tended to increase with decreasing nodule size. On the other hand, it was not observed in inter-reader 3D volume measurement. Therefore, the 3D software could help decrease the variations in volume measurements, especially when the measurements are performed by the same reader. For different readers, manual adjustments might lead to large variations, especially when the nodules are small. Thus, using a 3D computed semiautomatic software to measure lung nodules by different readers might reduce its reliability. A previous study reported that there was relatively good agreement among nine consecutive measurements made by three independent readers [27] using the GE lung analysis software. In the present study, there was a larger number of juxta-structure nodules (63%, 52 of 83), which may have highly irregular shapes.

The analysis of nodule types suggested that the isolated nodule had smaller variations in size/volume than the other types. The juxta-vascular type showed the largest variations in linear measurement and calculated volume measurement. Nevertheless, the measurement variation of the juxta-vascular type could be reduced using 3D volume measurements performed by the same reader. Thus, adjacent structures might cause inaccurate manual measurements, but well-designed 3D semi-automatic software can help identify the boundaries between nodules and adjacent structures effectively. A previous phantom study on the effects of blood vessels on nodule volume measurement reported a partially similar finding [28]; it indicated that the presence of adjacent vessels might produce the linear measurement errors. This difference probably results from the presence of the pulmonary structures with attenuation values like pulmonary nodules, and that may extend or reduce the segmentation range from the background. Although recent studies examined isolated, juxta-vascular, and juxta-pleural nodules, the variability in volume measurements has not been thoroughly investigated for the juxta-structure nodules [9,15,29,30].

Although inter- and intra-reader variability in nodule volume measurement has been extensively studied [29,30,31,32], the intra-reader variability varied among studies. Erasmus [32] reported that the inter-reader variability of diameter measurements was greater than that of intra-reader measurement. Revel et al. [33] reported poor inter-reader reliability in diameter measurements. In lung cancer, Wang et al. [34] reported that the intra-observer agreement was satisfying, while the inter-observer agreement was limited. The results reported here delineated a great variability between intra-reader and inter-reader comparisons. The inter-reader variation was always larger than that of the intra-reader comparison. The 3D volume measurement showed the smallest variation compared with other methods in the intra-reader comparison, while this was not that prominent in the inter-reader comparison. The reason might be that the 3D software used here was semiautomatic, and the variations in the determination of the nodule center and the manual adjustments of the nodule boundaries by different readers were significant.

The present study had limitations. The true values of the diameter and volume of the pulmonary nodules were unknown because the nodules were not removed in many cases. Measurement precision is the most important aspect of pulmonary nodule volumetric analysis for assessing lesion growth with low-dose CT. Another limitation is that the 3D volumetric software seldom enables the segmentation of juxta-pleural or juxta-vascular nodules, although a visual control of the semiautomatic segmentation had to be used by overlaying the images with color-coded segmentation results.

## 5. Conclusions

Accurate measurement of the diameter and volume of pulmonary nodules is crucial for the diagnosis and follow-up strategy of pulmonary nodules. The present study indicates that 3D semiautomatic volume software shows a smaller variation than the calculated volume method using a linear measurement. Nodule size and location influence measurement variability. The variability among different readers in nodule volume measurement can be considerable. The results of the present study suggest that a 3D volume software is a better choice than the calculated volume, when possible. Baseline and follow-up measurements should be performed by the same reader whenever possible. For small and juxta-structure nodules, performing multiple measurements and calculating a mean value as the result could be a good method.

## Figures and Tables

**Table 1:** Descriptive statistics of the data obtained by the two readers.

Measurements	Reader A		Reader B
	A1	A2	
Longest diameter (mm)	6.4 ± 2.5 (3.4–18.3)	6.4 ± 2.6 (3.1–18.1)	6.3 ± 2.5 (3.0–18.0)
The calculated volume of the nodule (mm^3^)	222.6 ± 457.5 (20.6–3208.9)	224.7 ± 482.4 (15.6–3104.8)	212.9 ± 433.8 (14.1–3053.6)
3D semiautomatic volume (mm^3^)	86.8 ± 278.4 (4.89–2433.9)	86.7 ± 278.5 (4.9–2436.3)	83.5 ± 252.4 (4.4–2179.6)

Data are shown as means ± standard deviations. The numbers in parentheses are the ranges. A1 is the first measurement by reader A. A2 is the repeated measurement one week later by reader A.

**Table 2:** ICCs of overall measurement by size, type, and reader.

Measurement Comparisons		Overall	By Size(mm)			By Type		
			<6	≥6 and <8	≥8	Isolated	Juxta-Vascular	Juxta-Pleural
A1 *								
	c_volume-volume **	0.916	0.773	0.552	0.989	0.968	0.639	0.794
A2								
	c_volume-volume	0.900	0.739	0.542	0.966	0.981	0.635	0.721
B1								
	c_volume-volume	0.901	0.742	0.540	0.984	0.979	0.632	0.747
Overall								
	c_volume-volume	0.905	0.748	0.546	0.977	0.976	0.642	0.748

* A1 is the first measurement by reader A. A2 is the repeated measurement one week later by reader A. B1 is the first measurement by reader B. Overall is a summary of the relevant measurements. ** c_volume-volume means the intraclass correlation coefficient (ICC) of the calculated volume using the longest diameter and the 3D semiautomatic volume.

**Table 3:** Intra-reader and inter-reader measurements.

Measurement Comparisons	Overall	By Size (mm)			By Type		
		<6 mm	≥6 and<8 mm	≥8 mm	Isolated	Juxta-Vascular	Juxta-Pleura
Intra-reader							
A1diameter-A2 diameter *	0.997	0.980	0.977	0.994	0.998	0.995	0.997
A1 volume-A2 volume *	1.000	0.999	0.999	1.000	1.000	0.999	1.000
A1_c_volume-A2_c_volume *	0.992	0.983	0.984	0.988	0.994	0.999	0.991
Inter-reader							
A1 diameter-B1 diameter	0.995	0.941	0.942	0.996	0.995	0.989	0.996
A1volume-B1 volume	0.994	0.987	0.987	0.994	1.000	0.986	0.993
A1_c_volume-B1_c_volume	0.998	0.944	0.958	0.997	0.986	0.992	0.999

* A1 diameter-A2 diameter means the intraclass correlation coefficient (ICC) of the longest diameters of the lung nodules between the first (A1) and second (A2) measurements by reader A. A1 volume-A2 volume means the ICC for the 3D semiautomatic volume measurements between the first (A1) and second (A2) measurements by reader A. A1_c_volume-A2_c_volume means the ICC for the calculated volume using the longest diameter between the first (A1) and second (A2) measurements by reader A. B1 is the first measurement by reader B.

**Table 4:** Mean differences and limits of agreement of overall measurement.

Measurement Comparisons	Mean Difference % (95% CI)	95% Limits of Agreement
Intra-reader		
A1 diameter -A2 diameter *	−0.5 (−1.13, 0.13)	−6.2, 5.2
A1 volume-A2 volume *	0.2 (−0.25, 0.73)	−4.1, 4.6
A1_c_volume-A2_c_volume *	−1.5 (−3.36, 0.39)	−18.3, 15.3
Inter-reader		
A1 diameter -B1 diameter	−1.5 (−2.48, −0.44)	−10.6, 7.7
A1 volume-B1 volume	−1 (−3.19, 1.25)	−20.9, 18.9
A1_c_volume-B1_c_volume	−4.3 (−7.35, −1.33)	−31.4, 22.7

Numbers in brackets are the 95% confidence intervals. * A1 diameter-A2 diameter means the intraclass correlation coefficient (ICC) of the longest diameters of the lung nodules between the first (A1) and second (A2) measurements by reader A. A1 volume-A2 volume means the ICC for the 3D semiautomatic volume measurements between the first (A1) and second (A2) measurements by reader A. B1 is the first measurement by reader B.

**Table 5:** Mean differences and limits of agreement of different nodule sizes.

Measurement Comparisons	<6 mm		≥6 and <8 mm		≥8 mm	
	Mean Difference % (95% CI)	95% Limits of Agreement	Mean Difference % (95% CI)	95% Limits of Agreement	Mean Difference % (95% CI)	95% Limits of Agreement
Intra-reader						
A1 diameter-A2 diameter	−1 (−2.03, 0.13)	−7.7, 5.8	0.2 (−0.57, 0.95)	−3.7, 4.1	−0.6 (−2.19, 0.97)	−5.7, 4.5
A1 volume-A2 volume	0.2 (−0.21, 0.68)	−2.5, 3.0	0 (−0.96, 0.91)	−4.8, 4.8	0.9 (−1.30, 3.02)	−6.1, 7.9
A1_c_volume-A2_c_volume	−2.8 (−6.03, 0.38)	−22.7, 17.1	0.6 (−1.71, 2.83)	−11.1, 12.3	−1.8 (−6.57, 2.90)	−17.2, 13.5
Inter-reader						
A1 diameter-B1 diameter	−2 (−3.90, −0.15)	−13.6, 9.6	−0.4(−1.61, 0.87)	−6.8, 6.0	−2.1 (−3.13, −1.02)	−5.5, 1.3
A1 volume-B1 volume	−0.7 (−4.04, 2.57)	−21.3, 19.8	−1.2 (−5.31, 2.94)	−22.4, 20.1	−1.2 (−6.02, 3.57)	−16.8, 14.3
A1_c_volume-B1_c_volume	−6 (−11.52, −0.51)	−40.2, 28.2	−1.1 (−4.82, 2.55)	−20.1, 17.9	−6.2 (−9.37, −3.05)	−16.5, 4.0

Numbers in brackets are the 95% confidence intervals. A1 diameter-A2 diameter means the intraclass correlation coefficient (ICC) of the longest diameters of the lung nodules between the first (A1) and second (A2) measurements by reader A. A1 volume-A2 volume means the ICC for the 3D semiautomatic volume measurements between the first (A1) and second (A2) measurements by reader A. A1_c_volume-A2_c_volume means the ICC for the calculated volume using the longest diameter between the first (A1) and second (A2) measurements by reader A. B1 is the first measurement by reader B.

**Table 6:** Mean difference and limits of agreement of different nodule types.

Measurement Comparisons	Isolated		Juxta-Vascular		Juxta-Pleural	
	Mean Difference % (95% CI)	95% Limits of Agreement	Mean Difference % (95%CI)	95% Limits of Agreement	Mean Difference % (95% CI)	95% Limits of Agreement
Intra-reader						
A1 diameter-A2 diameter *	−0.4 (−0.96, 0.26)	−3.6, 2.9	−1.5 (−3.52, 0.50)	−9.7, 6.7	−0.1 (−1.07, 0.96)	−5.7, 5.6
A1 volume-A2 volume *	0.9 (0.026, 1.78)	−3.8, 5.6	−0.3 (−0.98, 0.37)	−3.0, 2.4	−0.1 (−0.91, 0.77)	−4.7, 4.6
A1_c_volume-A2_c_volume *	−1.1 (−2.88, 0.77)	−10.8, 8.7	−4.5 (−10.41, 1.44)	−28.6, 19.6	−0.2 (−3.21, 2.87)	−17.0, 16.6
Inter-reader						
A1 diameter-B1 diameter	−2.1 (−3.24, −0.92)	−8.3, 4.1	−2.6 (−5.33, 0.10)	−13.7, 8.4	−0.2 (−2.01 1.61)	−10.2, 9.8
A1 volume-B1 volume	−0.8 (−4.20, 2.69)	−19.2, 17.6	−2.6 (−7.27, 1.97)	−21.4, 16.1	−0.2 (−4.21, 3.79)	−22.3, 21.9
A1_c_volume-B1_c_volume	−6.2 (−9.65, −2.76)	−24.6, 12.2	−7.7 (−15.66, 0.21)	−40, 24.5	−0.6 (−5.99, 4.70)	−30.2, 28.9

Numbers in brackets are the 95% confidence intervals. * A1 diameter-A2 diameter means the intraclass correlation coefficient (ICC) of the longest diameters of the lung nodules between the first (A1) and second (A2) measurements by reader A. A1 volume-A2 volume means the ICC for the 3D semiautomatic volume measurements between the first (A1) and second (A2) measurements by reader A. A1_c_volume-A2_c_volume means the ICC for the calculated volume using the longest diameter between the first (A1) and second (A2) measurements by reader A. B1 is the first measurement by reader B.

## Data Availability

Not applicable.
